# The Effects of Platycodin D, a Saponin Purified from *Platycodi Radix*, on Collagen-Induced DBA/1J Mouse Rheumatoid Arthritis

**DOI:** 10.1155/2014/954508

**Published:** 2014-01-06

**Authors:** O. Gon Kwon, Sae Kwang Ku, Hee Duk An, Young Joon Lee

**Affiliations:** ^1^Department of Rehabilitation Medicine, College of Korean Medicine, Daegu Haany University, 1 Hannydaero, Gyeongsan, Gyeongsangbuk-Do 712-715, Republic of Korea; ^2^The Medical Research Center for Globalization of Herbal Medicine, College of Korean Medicine, Daegu Haany University, 1 Hannydaero, Gyeongsan, Gyeongsangbuk-Do 712-715, Republic of Korea; ^3^Department of Histology and Anatomy, College of Korean Medicine, Daegu Haany University, 1 Hannydaero, Gyeongsan, Gyeongsangbuk-Do 712-715, Republic of Korea; ^4^Department of Preventive Medicine, College of Korean Medicine, Daegu Haany University, 1 Hannydaero, Gyeongsan, Gyeongsangbuk-Do 712-715, Republic of Korea

## Abstract

The object of this study is to observe the effects of platycodin D, a saponin purified from *Platycodi Radix*, on mice collagen-induced arthritis (CIA). A daily dose of 200, 100, and 50 mg/kg platycodin D was administered orally to male DBA/1J mice for 40 days after initial collagen immunization. To ascertain the effects administering the collagen booster, CIA-related features (including body weight, poly-arthritis, knee and paw thickness, and paw weight increase) was measured from histopathological changes in the spleen, left popliteal lymph node, third digit, and the knee joint regions. CIA-related bone and cartilage damage improved significantly in the platycodin D-administered CIA mice. Additionally, myeloperoxidase (MPO) levels in the paw were reduced in platycodin D-treated CIA mice compared to CIA control groups. The level of malondialdehyde (MDA), an indicator of oxidative stress, decreased in a dose-dependent manner in the platycodin D group. Finally, the production of IL-6 and TNF-**α**, involved in rheumatoid arthritis pathogenesis, was suppressed by treatment with platycodin D. Taken together, these results suggest that platycodin D is a promising new effective antirheumatoid arthritis agent, exerting anti-inflammatory, antioxidative and immunomodulatory effects in CIA mice.

## 1. Introduction

Rheumatoid arthritis (RA) is a common human autoimmune disease characterized by chronic inflammation of the synovial membranes with concomitant destruction of cartilage and bone. Although the etiology and pathogenesis of RA are not yet understood, it has been suggested that abnormalities of cytokines, such as interleukin (IL)-1, IL-6, and TNF-*α*, play an important role in the pathogenesis [1]. In addition, the current view of the cytokine network in rheumatoid joints supports the notion that TNF-*α* activates a cytokine cascade characterized by the simultaneous production of proinflammatory cytokines such as IL-1 and IL-6 and of anti-inflammatory cytokines such as IL-10, IL-1Ra, and soluble TNF receptor [2]. In epidemiological studies, oxidative damage to proteins, lipids, DNA, cartilage, and extracellular collagen has been demonstrated in patients with RA [3] and moreover has demonstrated an inverse correlation between the dietary intake of antioxidants and the incidence of RA [4]. Lipid peroxidation markers such as serum malondialdehyde (MDA) and urine isoprostane are reported to be elevated in collagen-induced arthritis (CIA) compared with those in controls [5, 6]. As above concepts for pathogenesis of RA, clinical application with TNF-*α*-neutralizing antibody and IL-1 receptor antagonist exhibits substantial efficacy but carries the disadvantages of high cost, hypersensitivity to medications, and possibility of serious infections [7, 8]. In addition, antioxidants also showed favorable effects on the RA but their effects are much lower than the expectation [9, 10]. Therefore, further efforts are necessary to develop new drugs with fewer side effects and much more potent for treatment of RA.

As increase of the concern in the functional food and wellbeing in life, the demands and consumption of functional food originated from natural sources are increased [12]. *Platycodi Radix*, the roots of *Platycodon grandiflorum* (Jacq.), has been used traditionally as an expectorant and a remedy for bronchitis, tonsillitis, laryngitis, and suppurative dermatitis in China, Korea, and Japan. In China and Korea, the fresh roots of *P. grandiflorum* have been eaten as pickles for preventing obesity [13]. Platycodin D is a major pharmacological constituent of *Platycodi Radix* [14], and it has been showen the antidiabetic [14–16], anti-inflammatory [17–19], anticancer [20, 21], antinociceptive [22, 23], and immunomodulatory [24, 25] activities.

The object of this study was, therefore, to evaluate the efficacy of platycodin D, a saponin purified from *Platycodi Radix* on mice CIA. In the present study, 50, 100, and 200 mg/kg of platycodin D were orally administered to male DBA/1J mice for 40 days, once a day from the initial collagen immunization. The changes on the body weight, clinical scores, thicknesses of left knee and paw, spleen, left popliteal lymph node and left hid paw weights, paw myeloperoxidase (MPO; for neutrophil infiltration) and malondialdehyde (MDA; for oxidative stress) contents, paw TNF-*α* and interleukin (IL)-6 levels, splenocytes TNF-*α* and IL-6 productions and histopathology of spleen, left popliteal lymph node, third digits, and knee joint regions were monitored using established methods. Nonimmunized and nonboosted mice were used as a normal control, and Enbrel, TNF-*α* neutralizing antibody, was used as reference substances in this study.

## 2. Materials and Methods

### 2.1. Animals and Husbandry

Forty-eight males DBA/1JJmsSlc (5-week-old upon receipt; SLC, Japan) were used after acclimatization for 14 days. Animals were housed four or five per polycarbonate cage in a temperature (20–25°C)—and humidity (40–45%)—controlled room with a 12 hrs : 12 hrs light : dark cycle. Feed (Samyang, Korea) and water were supplied *ad libitum*. All animals were fasted overnight before at immunization and sacrifice (about 18 hrs with *ad libitum* access to water) and treated according to the Guide for the Care and Use of Laboratory Animals [26]. In this study, eight mice per groups, total 6 groups, were divided.

### 2.2. Preparations and Administration of Test Materials

**Figure 1:**
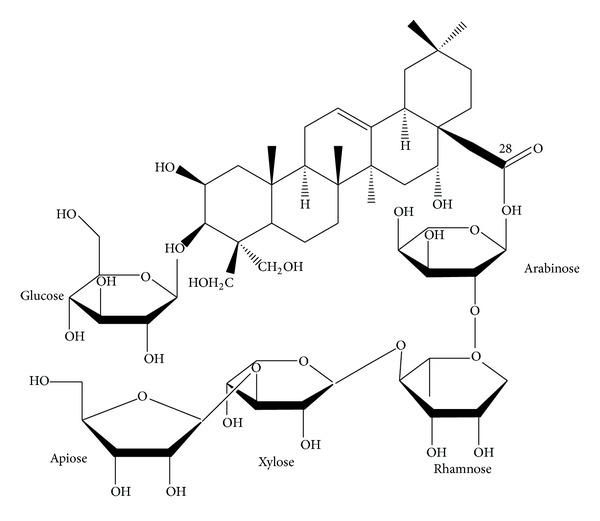
Chemical structure of platycodin D used in this study. Platycodin D is a triterpenoid bidesmoside, composed of an aglycone moiety, 3-Glc, and 28-O-Api-Xyl-Rha-Ara [11].

The platycodin D, gift from Glucan Corp. Ltd (Korea), was extracted from *Platycodi Radix* by previous method [11]. The raw sample (100 kg) of platycodin radix was extracted with methanol and partitioned sequentially with n-hexane, chloroform, ethyl acetate, and n-butanol. The n-butanol fraction was then subjected to Diaion HP-20 resin (Mitsubishi, Japan), and the fractions eluted at 60–80% of methanol were collected to obtain 90 g of crude saponins. The crude saponins were further purified by repeated silica gel (Merck, Germany) chromatography to obtain the purified platycodin D. The process was repeated several times until a sufficient quantity of platycodin D was obtained. The purified platycodin D was identified on the basis of Rf, FAB-MS (=1225.38), and [_13_C]-NMR spectra compared with the authentic platycodin D (Figure 1). Prepared platycodin D is light yellow powder and is stored in a desiccator to be protected from light and humidity. Platycodin D is well dissolved (clear light yellow solution) at least 40 mg/mL concentrations in distilled water. Enbrel (Wyeth Korea, Korea) 25 mg/0.5 mL vehicle packed in syringe was purchase from local supplier.

In this study, we selected 200 mg/kg of platycodin D as the highest dosage, and 100 and 50 mg/kg were selected as the middle and lowest dosages using common ratio 2. In addition, 10 mg/kg Enbrel, TNF-*α* neutralizing antibody, (injected three day-intervals) was used as reference drug. In the present study, 200, 100, and 50 mg/kg of platycodin D were orally administered, once a day 40 days in a volume of 5 mL/kg of distilled water. In case of Enbrel, it subcutaneously injected three day-intervals from immunization to sacrifice diluted using saline at 10 mg/kg levels. In intact and CIA control, only distilled water was orally administered instead of test materials, once a day for 40 days from immunization.

### 2.3. Induction of CIA

CIA was induced according to the previous methods [27]. Mice were immunized intradermally at the base of tail with 100 *μ*g of type II collagen (Chondrex, WA, USA) emulsified with an equal volume of Freund's complete adjuvant (Chondrex, WA, USA) and intraperitoneally boosted with type II collagen (100 *μ*g in 0.05 M acetic acid) emulsified with an equal volume of Freund's incomplete adjuvant (Chondrex, WA, USA) at 23 days after the initial immunization. In intact control mice, only vehicle, 0.05 M acetic acid was intradermally injected instead of collagen immunization and intraperitoneally administered instead of antigen boosting, respectively.

### 2.4. Changes in Body Weights

Changes of body weight were calculated at 1 day before immunization (Day −1), at immunization (Day 0), 1, 7, 14, 21, 23 (at antigen boosting), 28, 35, and 39 days after immunization with at a termination using an automatic electronic balance (Precisa Instrument, Switzerland). At immunization (initiation of administration) and at a termination, all experimental animals were overnight fasted (water was not; about 12 hr) to reduce the differences from feeding.

### 2.5. Estimation of Clinical Arthritis Indexes in CIA

The clinical severity of arthritis in all four paws of mice was evaluated in a triple-blind fashion by a previous published scoring system [28]. Briefly, 0: normal; 1: mild, apparent swelling limited to individual digits; 2: moderate, redness, and swelling of the ankle; 3: redness and swelling of the paw including digits; and 4: maximally inflamed limb with involvement of multiple joints. The arthritis score for each mouse was the sum of four paws, with the highest score of 16 for each mouse and recorded at antigen boosting (Day 23), 24, 25, 26, 27, 30, 33, 36, 39, and 40 days after immunization.

### 2.6. Paw and Knee Thickness Measurement

The thickness of left hind paw and knee was measured using an electronic digital caliper (Mitutoyo, Japan) and recorded at antigen boosting (Day 23), 24, 25, 26, 27, 30, 33, 36 and 39 days after immunization. In addition, to reduce the individual differences from start of measurements, the changes after antigen challenges were also calculated.

### 2.7. Organ Weight Measurements

At sacrifice, the spleen, left hind paw and left popliteal lymph nodes were collected after connective tissues, muscles, and debris were removed. The weight of organs or paw was calculated at g levels regarding absolute wet-weights. To reduce the individual body weight differences, the relative weight (%) was calculated using body weight at sacrifice and absolute weight.

### 2.8. Measurement of MPO Contents

Collected paws were homogenized (50 mg/mL) in 0.5% hexadecyltrimethylammonium bromide (Sigma, MO, USA) in 10 mM 3-N-morpholinopropanesulfonic acid (Sigma, MO, USA) and centrifuged at 15,000 g for 40 min. The suspension was then sonicated three times for 30 seconds. An aliquot of supernatant (20 *μ*L) was mixed with a solution of 1.6 mM tetra-methyl-benzidine (Wako, Japan) and 1 mM hydrogen peroxide (Daejung, Korea). Activity was measured spectrophotometrically as the change in absorbance at 650 nm at 37°C, using a microplate reader [29]. Results are expressed as milliunits (mU) of MPO activity per mg of protein, which were determined with the Bradford assay [30].

### 2.9. Measurement of MDA Contents

MDA formation was utilized to quantify the lipid peroxidation in the mouse paws and measured as thiobarbituric acid-reactive material. Tissues were homogenized (100 mg/mL) in 1.15% KCl buffer. Two hundred microliters of the homogenates were then added to a reaction mixture consisting of 1.5 mL 0.8% thiobarbituric acid (Sigma, MO, USA), 200 *μ*L 8.1% sodium dodecyl sulfate (Wako, Japan), 1.5 mL 20% acetic acid (pH 3.5), and 600 *μ*L distilled water. The mixture was then heated at 90°C for 45 min. After cooling to room temperature, the samples were cleared by centrifugation (10,000 g for 10 min) and their absorbance measured at 532 nm, using 1,1,3,3-tetramethoxypropane as an external standard [29]. The level of lipid peroxides was expressed as nmol of MDA/mg of protein.

### 2.10. Measurement of Paw Cytokine Contents

Paws were snap frozen in liquid nitrogen; the sample was then homogenized in 700 *μ*L of a TRIS-HCl buffer containing protease inhibitors. Samples were centrifuged for 30 min and the supernatant was frozen at −80°C until assay. Cytokine levels were determined using commercial ELISA kit [31].

### 2.11. Measurement of Splenocyte Cytokine Productions

Mouse spleens were collected for cell preparation and washed twice with PBS. The spleens were minced and the red blood cells were lysed with 0.83% ammonium chloride (Daejung, Korea). The cells were filtered through a cell strainer and centrifuged at 1300 rpm at 4°C for 5 min. The cell pellets were resuspended in RPMI 1640 medium and plated in 24-well plates (Corning, NY, USA) at a concentration of 1 × 10^6^ cells/well. Isolated splenocytes were cultured for 72 h. The amounts of TNF-*α* and IL-6 in the culture supernatants were measured by ELISA [32]. The amounts of cytokines present in the test samples were determined from standard curves constructed with serial dilutions of recombinant murine TNF-*α* and IL-6. The absorbance was determined with an ELISA microplate reader at 405 nm.

### 2.12. Histopathology

The knee joints parts were sampled with the joint capsules preservation with total left hind paw and fixed in 10% neutral buffered formalin. After 5 days of fixation, they were decalcified using decalcifying solution (24.4% formic acid and 0.5 N sodium hydroxide) for 5 days (mixed decalcifying solution was exchanged once a day for 5 days). After that, median joint parts were longitudinally trimmed or third digits were crossly trimmed, and embedded in paraffin, sectioned (3~4 *μ*m) and stained with hematoxylin and eosin (H&E). The histological profiles of the knee joints and third digits were observed as compared with the intact control. In addition, spleen and left popliteal lymph nodes were sampled and fixed in 10% neutral buffered formalin. After paraffin embedding, 3-4 *μ*m sections were prepared. Representative sections were stained H&E for light microscopical examination. After that the histological profiles of individual organs were observed.

*Histomorphometry.* The thickness of articular surface (including compact bone and articular cartilages) of tibia and femur (*μ*m/bone), articular cartilage thickness of tibia and femur (*μ*m/bone), infiltrated inflammatory cell numbers of left knee cavity and cutaneous regions of third digits (cells/mm^2^), dorsum digit skin thicknesses around third digits (*μ*m/digit), and cortical bone thickness of third digits (*μ*m/digit) were measured as histomorphometrical analyses at prepared knee joint and third digit samples for RA analysis using digital image analyzer (DMI-300, DMI, Korea). In addition, total (central regions; mm/spleen) and white pulp (mm/pulp) thicknesses of spleen with numbers of white pulp (pulps/mm^2^) and total (central regions) and cortex thicknesses (mm/lymph node) of left popliteal lymph were measured as histomorphometrical analyses for immune response at prepared spleen and thymus samples using digital image analyzer according to the previous report [33]. The histopathologist was blinds to group distribution when this analysis was made.

### 2.13. Statistical Analyses

Multiple comparison tests for different dose groups were conducted. Variance homogeneity was examined using the Levene test. If the Levene test indicated no significant deviations from variance homogeneity, the obtained data were analyzed by one way ANOVA test followed by least-significant differences (LSD) multicomparison test to determine which pairs of group comparison were significantly different. In case of significant deviations from variance homogeneity being observed at Levene test, a nonparametric comparison test, Kruskal-Wallis H test was conducted. When a significant difference is observed in the Kruskal-Wallis H test, the Mann-Whitney *U* (MW) test was conducted to determine the specific pairs of group comparison, which are significantly different. Statistical analyses were conducted using SPSS for Windows (Release 12.0K, SPSS Inc., USA).

## 3. Results

### 3.1. Changes in Body Weight

**Figure 2:**
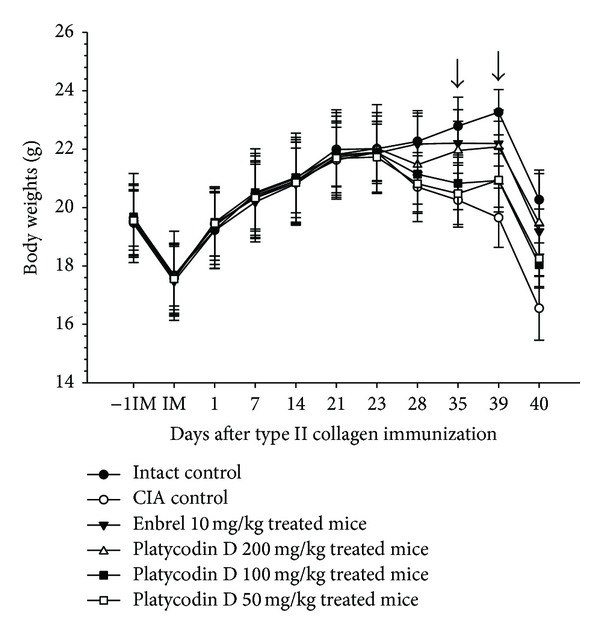
Platycodin D inhibited changes in body weight in CIA mice. Values are expressed as means ± SD (*n* = 8); platycodin D 200 mg/kg treated mice showed significant (*P* < 0.01) increases of body weight from 35 days after treatment, platycodin D 100 and 50 mg/kg treated mice showed significant (*P* < 0.01) increases of body weight from 39 days after treatment, respectively (dot arrow). CIA, collagen-induced arthritis; −1IM, 1 day before immunization; IM, immunization (start of administration of test materials); 23, day of antigen challenge. All animals were fasted overnight before immunization and sacrifice (40 days after immunization).

Significant decreases of body weight were detected from 28 days after immunization (5 days after antigen boosting) in CIA control as compared with intact control. Body weight gains were also significantly decreased after antigen boosting and during experimental periods. However, the body weight of Enbrel mice and mice treated with the three dosages of platycodin D increased markedly from 28 days after immunizations, as did the body weights after antigen boosting and during experimental periods (Figure 2).

### 3.2. Therapeutic Effects on Rheumatoid Arthritis

**Figure 3:**
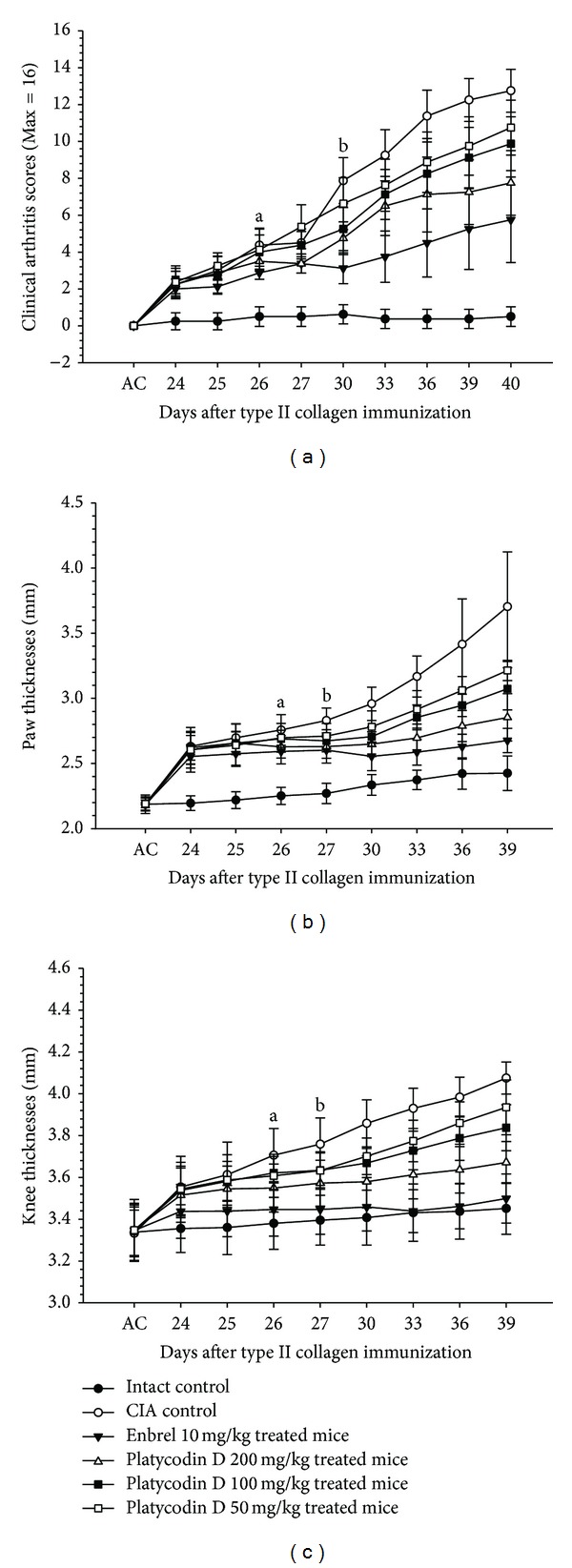
Platycodin D reduced clinical arthritis scores and paw and knee thickness in platycodin D-treated CIA mice. (a) Decreases of clinical arthritis scores were detected in platycodin D treated mice as compared with CIA control mice. (b) Paw thickness was reduced in platycodin D treated mice as compared with CIA control mice. (c) Knee thickness was reduced in platycodin D treated mice as compared with CIA control mice. Values are expressed as means ± SD (*n* = 8); ^a^ *P* < 0.05 in platycodin D 200 mg/kg treated mice, ^b^in platycodin D 100 and 50 mg/kg treated mice as compared with CIA control mice. AC: day of antigen challenge at 23 days after immunization. All animals were fasted overnight before immunization.

Clinical arthritis scores were significantly higher from 24 days after immunization (1 day after antigen boosting) in CIA control compared with intact control. However, significant decreases in clinical arthritis scores were detected from 25 days after immunization in Enbrel, from 26 days after immunization in platycodin D 200 mg/kg treated mice, and from 30 days after immunization in platycodin D 100 and 50 mg/kg treated mice as compared with CIA control mice, respectively (Figure 3(a)).

There were a significant increases in paw thicknesses and knee thickness from 24 days after immunization (1 day after antigen boosting) in CIA control as compared with intact control. Paw and knee thickness after antigen challenges were also increased significantly.

Paw thickness in Enbrel and 200 mg/kg platycodin D treated mice were decreased significantly from 25 and 26 days after immunizations, respectively. Mice treated with 100 and 50 mg/kg platycodin D also exhibited decreased paw thickness from 27 days after immunization. In addition, after antigen challenge, paw thickness decreased in Enbrel group, and in all three platycodin D-treated groups, as compared with the CIA control (Figure 3(b)).

Knee thickness in Enbrel and 200 mg/kg platycodin D treated mice decreased significantly from 25 and 26 days after immunizations, respectively, and from 27 days after immunization in 100 and 50 mg/kg platycodin D-treated mice as compared with CIA control mice. The changes on the knee thicknesses after antigen challenges were also significantly decreased in Enbrel and all three different dosages of platycodin D treated mice as compared with CIA control (Figure 3(c)).

The absolute and relative weights of the left paw, spleen, and left popliteal lymph nodes were significantly increases in CIA control, compared with the intact control. However, in the Enbrel treated group, and in all three different dosages of platycodin D treated groups, these weights were lower than in the CIA control group (Table 1).

### 3.3. Anti-Inflammatory and Immunomodulatory Effects of Platycodin D

**Figure 4:**
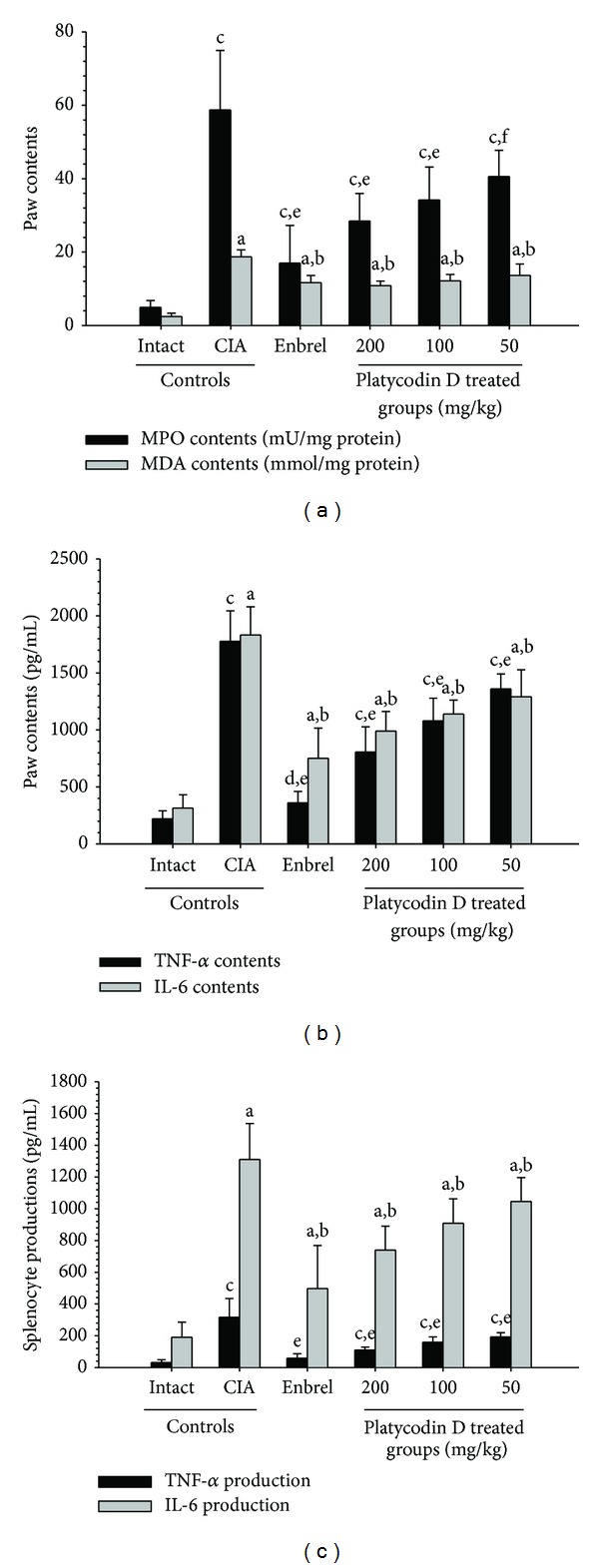
Platycodin D had a therapeutic effect on the CIA mice mediated by anti-inflammatory, antioxidative, and immunomodulatory effects. (a) Paw MPO and MDA contents of platycodin D-treated mice were lower compared to CIA control mice. (b) Paw TNF-*α* and IL-6 levels in platycodin D-treated mice were lower compared with CIA control mice. (c) Splenocyte TNF-*α* and IL-6 levels in platycodin D-treated mice were lower compared with CIA control mice. Values are expressed as means ± SD (*n* = 8); ^a^ *P* < 0.01 as compared with intact control by LSD test; ^b^ *P* < 0.01 as compared with CIA control by LSD test; ^c^ *P* < 0.01 and ^d^ *P* < 0.05 as compared with intact control by MW test; ^e^ *P* < 0.01 and ^f^ *P* < 0.05 as compared with CIA control by MW test.

MPO and MDA contents in right hind paw were higher in CIA control as compared with intact control at sacrifice, respectively. However these paw MPO and MDA contents of Enbrel and all three different dosages of platycodin D treated group were significantly decreased as compared with CIA control mice, respectively (Figure 4(a)).

There was significant increment of TNF-*α* and IL-6 contents in right hind paw were detected in CIA control as compared with intact control at sacrifice. In addition, splenocyte TNF-*α* and IL-6 productions increased in the CIA control compared with the intact control.

However, for the Enbrel group, and all three different dosages of platycodin D treated group were significantly lower as compared with CIA control mice (Figures 4(b) and 4(c)).

### 3.4. Histopathological Changes in the Knee and Third Digits

**Figure 5:**
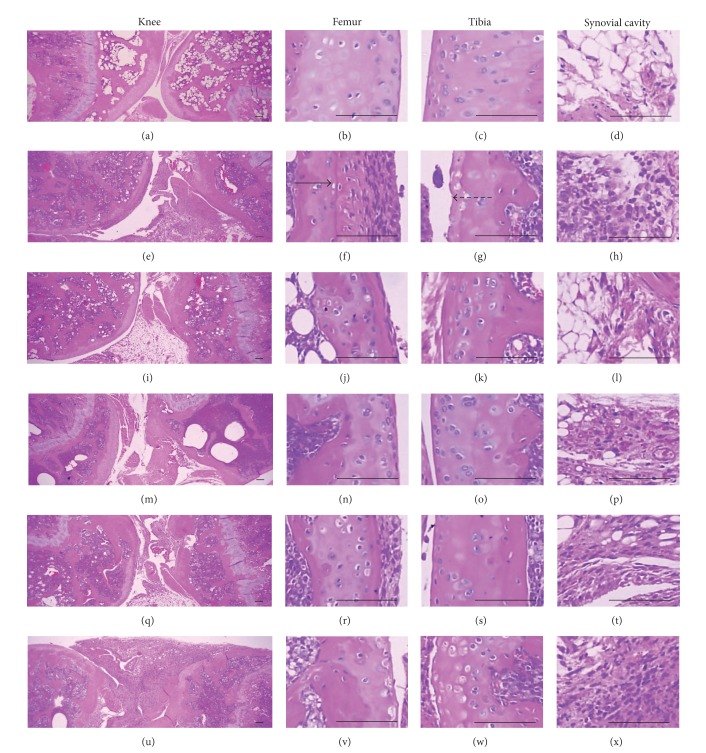
Histopathological profiles of the knee in the intact control ((a)–(d)), CIA control ((e)–(h)), Enbrel group ((i)–(l)), platycodin D-treated groups; 200 mg/kg ((m)–(p)), 100 mg/kg ((q)–(t)), and 50 mg/kg ((u)–(x)). Marked decreases in the articular surfaces (cartilage and bone) were detected in the knee articular surfaces of the femur and tibia, with severe inflammatory cell infiltration into the synovial cavity in the CIA control mice. However, histopathological changes in the CIA group were decreased dramatically by treatment with platycodin D. The arrow indicates articular surface thickness. Dotted arrow indicates articular cartilage thickness. All were stained with H&E. Scale bars = 160 *μ*m.

**Figure 6:**
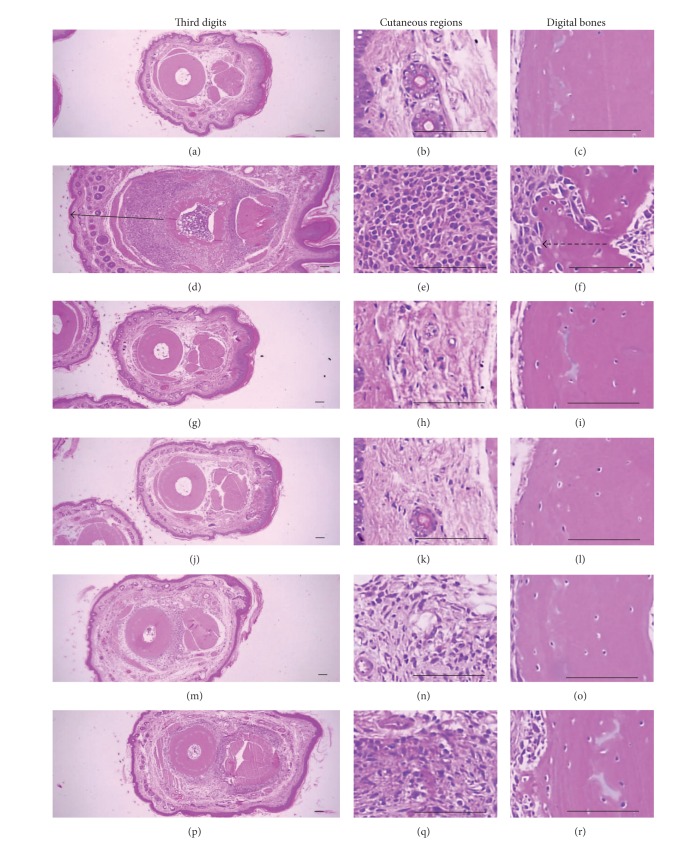
Histopathological profiles of the third digits in the intact control ((a)–(c)), CIA control ((d)–(f)), Enbrel group ((g)–(i)), platycodin D-treated groups; 200 mg/kg ((j)–(l)), 100 mg/kg ((m)–(o)), and 50 mg/kg ((p)–(r)). Marked edematous changes, inflammatory cell infiltration, and erosive damage of digital bones were detected on the third digits of the CIA control mice. However, these histopathological changes were decreased dramatically by treatment with platycodin D. The arrow indicates the dorsum paw skin thickness. Doted arrow indicates cortical bone thickness. All were stained with H&E. Scale bars = 160 *μ*m.

Marked decreases in articular cartilages and bones surfaces were detected in both knee articular, femur, and tibia surfaces, where there was marked inflammatory cell infiltration into the synovial cavity in the CIA control mice. There were also dramatic edematous changes, inflammatory cell infiltrations, and erosive damages of digital bones on the third digits of the CIA control mice. However, these classic histopathological changes indicative of CIA were dramatically decreased by treatment of Enbrel and all three different dosages of platycodin D, respectively (Figures 5 and 6).

**Figure 7:**
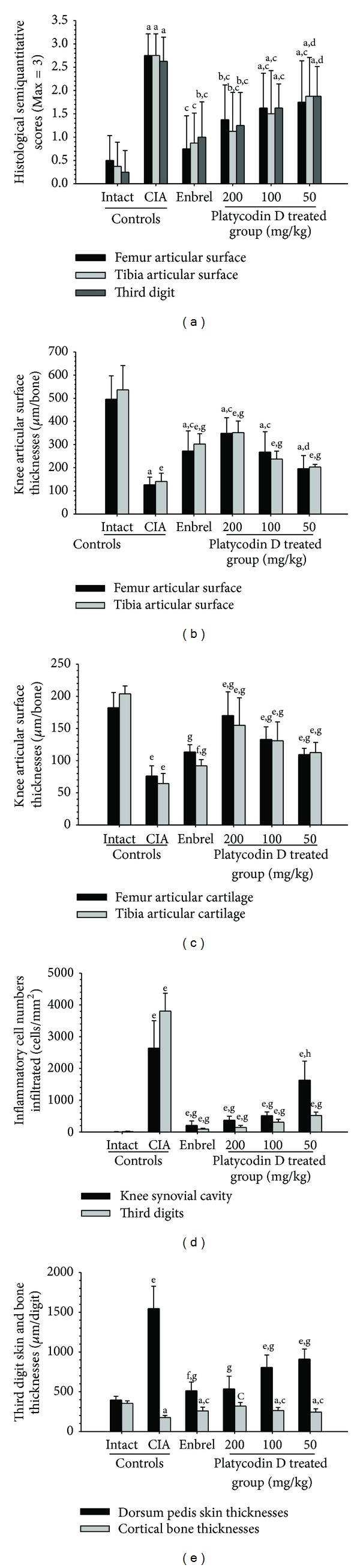
Platycodin D ameliorated the histopathological changes of the knee and third digit. (a) The semiquantitative scores of platycodin D-treated mice were lower compared with the CIA control mice. (b) The knee articular surface thickness of platycodin D-treated mice was greater compared with the CIA control mice. Knee articular surface thicknesses are shown in Figure 5 (arrow). (c) The knee articular cartilage thickness of platycodin D-treated mice was greater compared with the CIA control mice. Knee articular cartilage thicknesses are shown in Figure 5 (dotted arrow). (d) Infiltrated inflammatory cells of platycodin D-treated mice were lower compared with the CIA control mice (e). Increases in the third digit dorsum pedis skin thickness and decreases in the cortical bone thickness were inhibited by treatment with platycodin D compared with the CIA control mice. Measurements of the third digit dorsum pedis skin (arrow) and cortical bone (dotted arrow) thicknesses are shown in Figure 6. Values are expressed as means ± SD (*n* = 8); ^a^ *P* < 0.01 and ^b^ *P* < 0.05 as compared with intact control by LSD test; ^c^ *P* < 0.01 and ^d^ *P* < 0.05 as compared with CIA control by LSD test; ^e^ *P* < 0.01 and ^f^ *P* < 0.05 as compared with intact control by MW test; ^g^ *P* < 0.01 and ^h^ *P* < 0.05 as compared with CIA control by MW test.

The semiquantitative scores in the femur, tibia, and third digits of the CIA control group were increased significantly compared with the intact control. However these scores of Enbrel and all platycodin D treated mice were significantly decreased compared with CIA control mice (Figure 7(a)).

Knee articular surface thickness, including compact bone and cartilage, and knee articular cartilage thickness were significantly less in the femur and tibia of the CIA control compared with the intact control, whereas the thickness of these sites in the ENBREL and platycodin D-treated mice were markedly higher than in CIA control mice (Figures 7(b) and 7(c)).

The numbers of inflammatory cells infiltrated into the knee synovial cavity and third digits were significantly changed by platycodin D treatment. Significantly higher numbers of inflammatory cells infiltrated the knee synovial cavity and third digit cutaneous regions in the CIA control mice compared with the intact control; however, in the ENBREL and platycodin D-treated mice the numbers of infiltrated cells were lower compared with the CIA control mice (Figure 7(d)).

Significant increases of the third digit dorsum pedis skin and cortical thickness thicknesses were detected in platycodin D treated mice. The third digit dorsum pedis skin thicknesses was significantly higher in the CIA control; however there were significant decreases in the Enbrel, platycodin D-treated mice compared with the CIA control mice (Figure 7(e)).

### 3.5. Histopathological Changes of Secondary Lymphatic Organs

**Figure 8:**
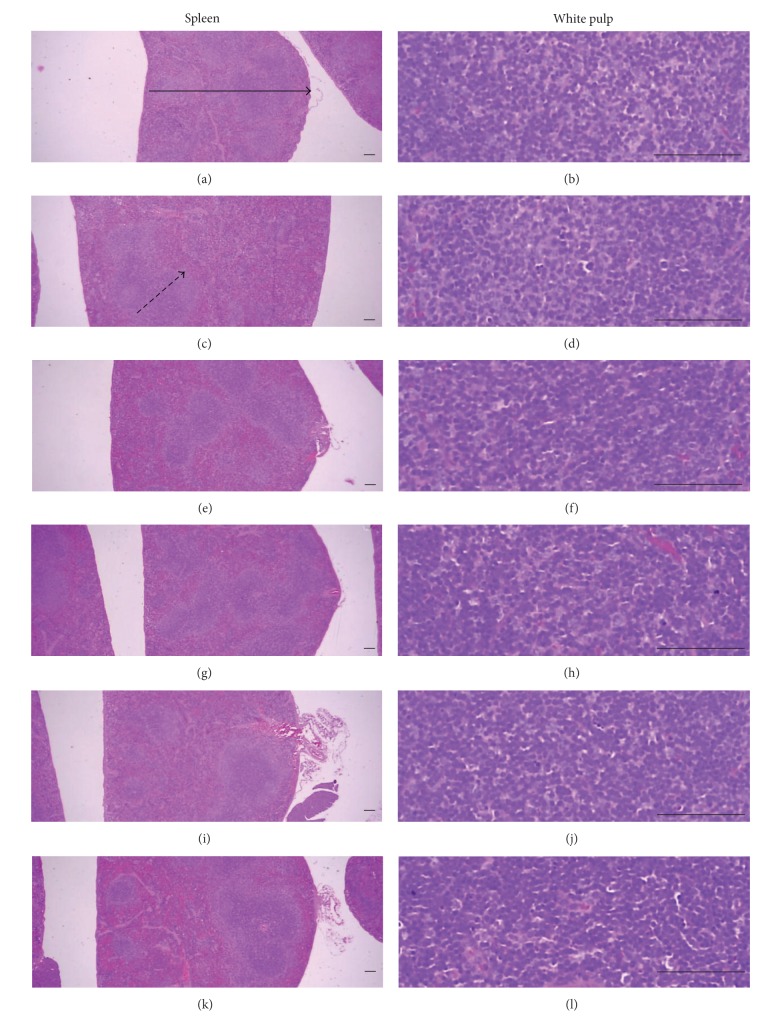
Histopathological profiles of the spleen in the intact control ((a), (b)), CIA control ((c), (d)), Enbrel group ((e), (f)), and platycodin D-treated groups; 200 mg/kg ((g), (h)), 100 mg/kg ((i), (j)), and 50 mg/kg ((k), (l)). Marked enlargement of the spleen, related to hyperplasia of lymphoid cells in the white pulp, was detected in the CIA control mice. However, these histopathological changes decreased dramatically after treatment with Enbrel and all three doses of platycodin D. Arrow indicates the total spleen thickness. Doted arrow indicates white pulp. All were stained with H&E. Scale bars = 160 *μ*m.

**Figure 9:**
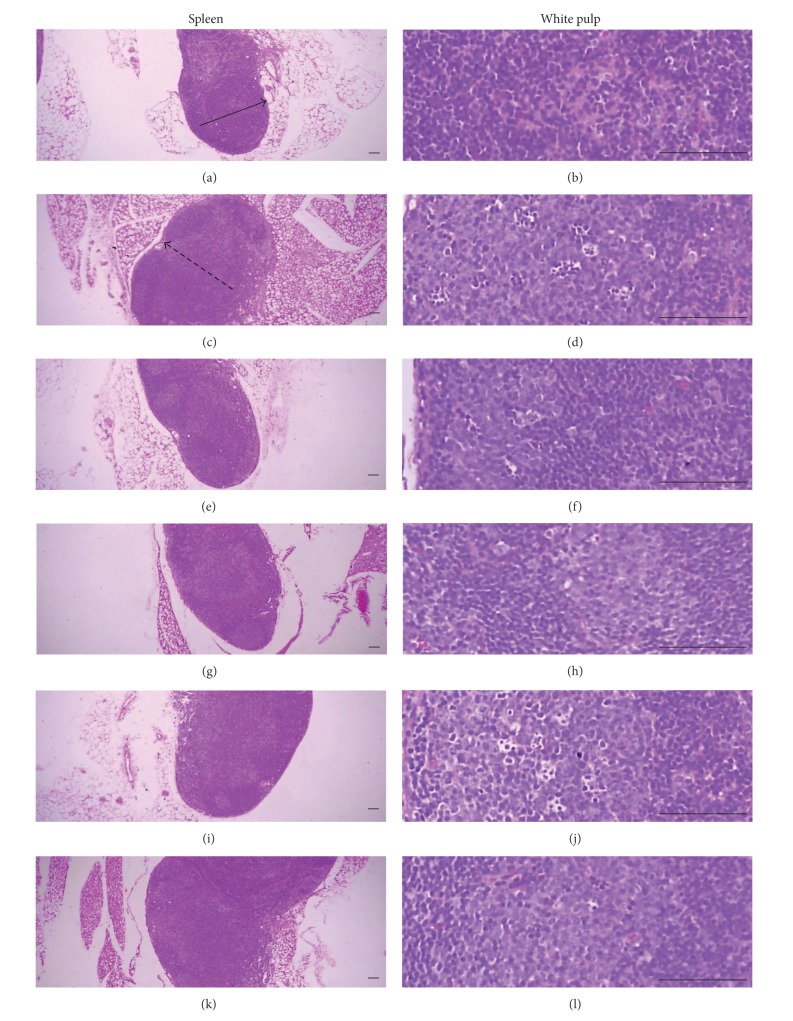
Histopathological profiles of the left popliteal lymph nodes in the intact control group ((a), (b)), CIA control group ((c), (d)), Enbrel group ((e), (f)), and platycodin D-treated groups; 200 mg/kg ((g), (h)), 100 mg/kg ((i), (j)), and 50 mg/kg ((k), (l)). Marked enlargement of popliteal lymph nodes, related to hyperplasia of lymphoid cells in the cortex of the lymph nodes, was detected in the CIA control mice. However, these histopathological changes decreased dramatically after treatment with Enbrel and all three doses of platycodin D. Arrow indicates the total popliteal lymph node thickness. Doted arrow indicates cortex thicknesses. All were stained with H&E. Scale bars = 160 *μ*m.

**Figure 10:**
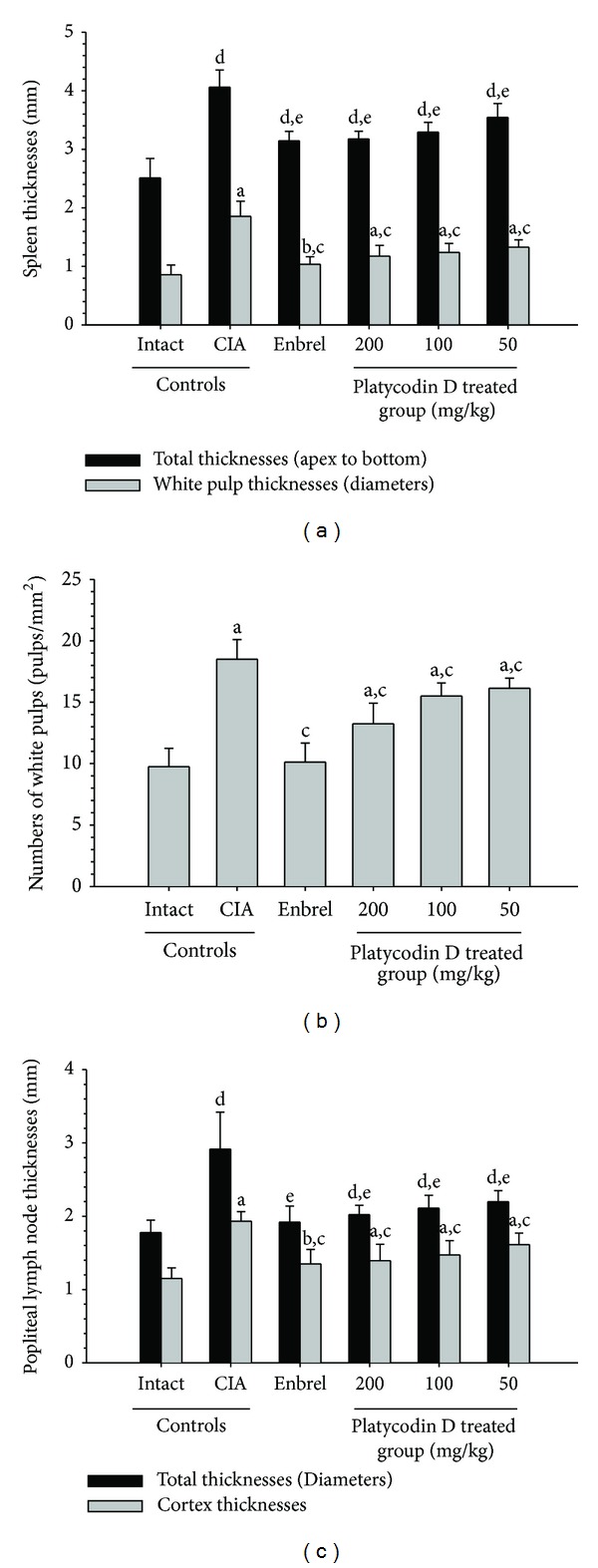
Platycodin D improved the histopathological changes of the secondary lymphatic organs. (a) The total spleen and white pulp thicknesses of platycodin D-treated mice were significantly decreased compared with the CIA control mice. Measurements of total spleen (arrow) and white pulp (dotted arrow) thicknesses are shown in Figure 8. (b) The splenic white pulp of platycodin D-treated mice was significantly lower compared with CIA control mice. (c) The popliteal lymph node total and cortex thickness of platycodin D-treated mice were significantly lower compared with the CIA control mice. Measurement of lymph node total (arrow) and cortex (dotted arrow) thicknesses are shown in Figure 9. Values are expressed as means ± SD (*n* = 8); ^a^ *P* < 0.01 and ^b^ *P* < 0.05 as compared with intact control by LSD test; ^c^ *P* < 0.01 as compared with CIA control by LSD test; ^d^ *P* < 0.01 as compared with intact control by MW test; ^e^ *P* < 0.01 as compared with CIA control by MW test.

Marked enlargement of spleen and popliteal lymph nodes were detected in CIA control mice. It is related to hyperplasia of lymphoid cells in the white pulp of spleen or cortex of lymph nodes. However, these histopathological changes of immune enhances were dramatically decreased by treatment of Enbrel and platycodin D (Figures 8 and 9). Also, total spleen thicknesses (around central regions; from apex to base), splenic white pulp numbers, splenic white pulp thickness, and popliteal lymph node total thickness were significantly lower in the Enbrel and platycodin D-treated mice compared with the CIA control mice (Figure 10).

## 4. Discussion

According to the results, platycodin D significantly improved CIA-related features, including poly-arthritis [34], body weight fluctuation [35, 36], knee and paw thickness, and paw weight increases [35]. These results suggested that platycodin D possesses an anti-inflammatory effect resulted from its antioxidant properties, since RA is a common human autoimmune disease characterized by chronic inflammation of the synovial membranes with concomitant destruction of cartilage and bone [1–6].

A marked reduction of body weights and gains have been detected in CIA mice after antigen exposures [35, 36], and significant decreases of body weights and gains were also detected in this study. However, these CIA related body weight and gain decreases were dose-dependently inhibited by treatment of platycodin D in this study. It is considered as one of the predictable evidence that platycodin D has favorable effects on the RA related body weight losses.

RA is characterized by the inflammation of synovial joints infiltrated by CD4+ T cells, macrophages, and plasma cells, neutrophils that play major roles in the pathogenesis of the disease [2, 37]. Besides their direct damaging effects on tissues, it is well established that oxygen metabolites play a role in the recruitment of neutrophils, preferentially polymorphoneutrophils (PMNs), into injured tissues [38]. Activated PMNs are also a potential source of oxygen metabolites [39] and MPO is one of activating cytotoxic enzymes released from PMNs [40]. In the present study, the increases of paw MPO levels observed in CIA control were marked inhibited by treatment of Enbrel or platycodin D. Therefore, it is considered that platycodin D has direct anti-inflammatory effects.

Inflammations were directly correlated with oxidative stress [41] and oxidative stresses are also involved in the pathogenesis of CIA [9, 10]. In addition, marked increases of MDA, a marker of lipid peroxidation were also reported in CIA paw [31]. In the present study, dramatically increases of paw MDA levels were also detected in CIA control mice. However, these increases of MDA contents were dose-dependently inhibited by treatment of platycodin D like Enbrel. These results suggested that platycodin D exerts anti-oxidant effect by inhibiting MDA.

Abnormal increases of cytokine, IL-6, and TNF-*α* have been involved in the pathogenesis of RA [1, 42]. The cytokine TNF-*α*, produced by a variety of cell types, including splenocytes, was found to be associated with critical events leading to T-lineage commitment and differentiation [43]. TNF-*α* can enhance the *in vivo* immune response at doses much lower than those that cause weight loss or tissue toxicity. It enhances proliferation of B and T cells and promotes the generation of cytotoxic T cells. In addition, it enhances IL-2-induced immunoglobulin production and augments IL-2 stimulated natural killer cell activity and proliferation of monocytes [44]. IL-6 has also been proposed to contribute to the development of arthritis [45]. IL-6 is known to be present at high levels in the serum and synovial fluid of RA and juvenile RA patients [46–48]. IL-6 acts as a stimulator of both B and T cell functions; it also promotes proliferation of plasmablastic precursors in the bone marrow and their final stage of maturation into immunoglobulin-producing plasma cells and participates in the activation and proliferation of T cells [45]. In the present study, marked increases of paw IL-6 and TNF-*α* contents were also observed with increases of splenocyte IL-6 and TNF-*α* productions. However, these increases of cytokine activities and productions were dose-dependently inhibited by treatment of platycodin D like Enbrel. These results are considered as one of indirect evidence that platycodin D has some immunomodulatory effects as already known [24, 25] and these immunomodulatory effects of platycodin D may involve the efficacies against the CIA detected in this study.

Clinical arthritis score system based on the gross edematous changes and inflammations have been used as a valuable scoring system to detect the efficacy of test materials on CIA [49]. In addition, poly-arthritis occurred in CIA [34, 50], and marked increases of paw and knee thicknesses were observed with significant increases of paw weights [35]. Histopathologically, inflammation of peripheral synovial joints including infiltrations of inflammatory cells, articular cartilage, and bone damages were induced in CIA, and semiquantitative score system based on these histopathological changes have been used as a valuable scoring system to detect the efficacy of test materials on CIA [32, 42, 49, 51, 52]. In the present study, severe inflammatory changes, cartilage, and bone erosions were observed on the knee and third hind digits of CIA control mice. Dose-dependent inhibition of these histopathological and histomorphometrical changes by treatment of platycodin D detected in the present study is considered as direct evidence that platycodin D has favorable effects on the CIA.

The importance of secondary lymphoid organs in the development of RA was demonstrated in early studies [53]. Generally, secondary lymphoid organs are enlarged due to hyperplasia of lymphoid cells [54, 55]. In the present study, enhanced immunity signs [33] as increases of spleen and lymph node weights, enlargement of spleen and lymph nodes due to hyperplasia of lymphoid cells were detected in CIA control at gross and histopathological observations. These enhanced immunities were dose-dependently inhibited by treatment of platycodin D like Enbrel.

## 5. Conclusion

The results obtained in this study suggest that oral treatment of platycodin D 200, 100, and 50 mg/kg showed relatively good favorable effects on the CIA mice mediated by anti-inflammatory, antioxidative, and immunomodulatory effects. These data suggest that platycodin D promise as a new effective antirheumatoid arthritis agent. Since marked favorable anti-CIA effects were also detected in platycodin D 50 mg/kg treated mice, the minimal effective dosage of platycodin D on CIA mice after oral administration is considered as below 50 mg/kg.

## Figures and Tables

**Table 1:** Changes of left hind paw and secondary lymphatic organs detected after treatment of Enbrel and three different dosages of platycodin D in CIA mice.

Groups	Paw weights		Spleen weights		Left popliteal lymph nodes weights	
	Absolute	Relative	Absolute	Relative	Absolute	Relative
Controls						
Intact	0.133 ± 0.008	0.667 ± 0.022	0.047 ± 0.008	0.236 ± 0.028	0.003 ± 0.002	0.013 ± 0.009
CIA	0.202 ± 0.027^a^	1.195 ± 0.188^a^	0.101 ± 0.009^a^	0.599 ± 0.075^a^	0.015 ± 0.004^a^	0.088 ± 0.025^a^
Reference						
Enbrel	0.136 ± 0.004^c^	0.665 ± 0.037^c^	0.062 ± 0.004^ac^	0.302 ± 0.022^ac^	0.005 ± 0.004^c^	0.025 ± 0.020^c^
Platycodin D						
200 mg/kg	0.128 ± 0.011^c^	0.688 ± 0.121^c^	0.055 ± 0.003^bc^	0.291 ± 0.022^ac^	0.007 ± 0.003^bc^	0.036 ± 0.017^bc^
100 mg/kg	0.140 ± 0.018^c^	0.763 ± 0.144^c^	0.062 ± 0.008^ac^	0.335 ± 0.047^ac^	0.008 ± 0.003^ac^	0.041 ± 0.015^ac^
50 mg/kg	0.154 ± 0.029^d^	0.902 ± 0.243^bd^	0.074 ± 0.019^ad^	0.435 ± 0.141^ad^	0.011 ± 0.003^ac^	0.061 ± 0.017^ac^

Values are expressed as mean ± SD, g (absolute weight), or % (relative weights versus body weights at sacrifice) of eight mice. ^a^ *P* < 0.01 as compared with intact control by LSD test, ^b^ *P* < 0.01 and ^c^ *P* < 0.05 as compared with CIA control by LSD test, and ^d^ *P* < 0.01 as compared with intact control by MW test.
